# Supplementary material for: Sorting at embryonic boundaries requires high heterotypic interfacial tension
Source: Nat Commun. 2017 Jul 31;8:157. doi: 10.1038/s41467-017-00146-x (PMC5537356; doi:10.1038/s41467-017-00146-x)
Supplement: Supplementary file 2 — Supplementary Software 1 [file 41467_2017_146_MOESM2_ESM.zip › PottsModel/SrcPottsModel/doc/model/ProtrusivePixel.html]

ProtrusivePixel


---


|  |  |  |
| --- | --- | --- |
| **Overview** \| **Package** \| **Class** \| **Use** \| **Tree** \| **Deprecated** \| **Index** \| **Help** | |  |
| **PREV CLASS**   **NEXT CLASS** | **FRAMES**    **NO FRAMES**     **All Classes** |  |
| SUMMARY: NESTED \| FIELD \| CONSTR \| METHOD | DETAIL: FIELD \| CONSTR \| METHOD |  |


---


## model Class ProtrusivePixel

```
java.lang.Object
  model.Pixel
      model.ProtrusivePixel
```

**All Implemented Interfaces:**: KnowsConstants

---

``` public class ProtrusivePixel extends Pixel ```

---

**Nested Class Summary**

**Nested classes/interfaces inherited from class model.Pixel**

`Pixel.PixelType`


| **Field Summary** | |
| --- | --- |
| `(package private)  boolean` | `aIsCenter` |
| `private static org.apache.logging.log4j.Logger` | `aLog` |
| `private static java.awt.Color` | `cTargetBoundaryPixelColor` |

**Fields inherited from class model.Pixel**

`aDisplay, aPositionManager, aX, aY, cHeatMapParam`


**Constructor Summary**

`ProtrusivePixel(int pX,
int pY,
Cell pC,
Lattice pL)`


| **Method Summary** | |
| --- | --- |
| `Pixel[]` | `copyTo(Pixel pTarget)`             Move pixel from one cell to the other and make appropriate changes. |
| `private  java.awt.Color` | `getAngleEnergyColor()` |
| `private  java.awt.Color` | `getAngleFromHorizontalColor()` |
| `double` | `getAngleWithTargetVector()` |
| `double` | `getAngleWithTargetVector(ProtrusiveCell c)` |
| `private  java.awt.Color` | `getAngleWithTargetVectorColor()` |
| `ProtrusiveCell` | `getCell()` |
| `java.awt.Color` | `getColor()` |
| `static double` | `getDeltaProtrusiveEnergy(ProtrusivePixel s, ProtrusivePixel t)` |
| `private  java.awt.Color` | `getHeadOrTailColor()` |
| `private static double` | `getHeadProtrusiveEnergy(double protrusionElasticity, double angle)` |
| `private static double` | `getTailProtrusiveEnergy(double protrusionElasticity, double angle)` |
| `protected  void` | `setAsCenter(boolean pOn)` |

**Methods inherited from class model.Pixel**

`adjustedBoundary, adjustedLocalEnergy, adjustedLocalTemperature, adjustedTimeInContact, areSwappable, atLatticeEdge, equals, getAllNeighbors, getAreaUnit, getCellBoundaryEdges, getCellType, getConnectedNeighbors, getConstants, getCoordinates, getDefaultPixelType, getDisplay, getDisplay, getDisplayCenterPoint, getEdgeUnit, getInformation, getLocalTemperature, getNumberOfNeighborsFromCell, getNumPixelEdges, getPixelInteraction, getPixelInteraction, getPositionManager, getRandomNeighbor, getType, getX, getY, hashCode, initialize, isBoundary, isInSameCell, isMedium, isNeighborWith, parametersToString, toString, updateParameters`

**Methods inherited from class java.lang.Object**

`clone, finalize, getClass, notify, notifyAll, wait, wait, wait`

**Field Detail**

### cTargetBoundaryPixelColor

```
private static java.awt.Color cTargetBoundaryPixelColor
```

---


### aIsCenter

```
boolean aIsCenter
```

---


### aLog

```
private static final org.apache.logging.log4j.Logger aLog
```


**Constructor Detail**

### ProtrusivePixel

```
public ProtrusivePixel(int pX,
                       int pY,
                       Cell pC,
                       Lattice pL)
```


**Method Detail**

### copyTo

```
public Pixel[] copyTo(Pixel pTarget)
```

:   **Description copied from class: `Pixel`**
:   Move pixel from one cell to the other and make appropriate changes.

    :   **Overrides:**: `copyTo` in class `Pixel`

---


### getHeadProtrusiveEnergy

```
private static double getHeadProtrusiveEnergy(double protrusionElasticity,
                                              double angle)
```

---


### getTailProtrusiveEnergy

```
private static double getTailProtrusiveEnergy(double protrusionElasticity,
                                              double angle)
```

---


### getDeltaProtrusiveEnergy

```
public static double getDeltaProtrusiveEnergy(ProtrusivePixel s,
                                              ProtrusivePixel t)
```

---


### getColor

```
public java.awt.Color getColor()
```

:   **Overrides:**: `getColor` in class `Pixel`

---


### getAngleFromHorizontalColor

```
private java.awt.Color getAngleFromHorizontalColor()
```

---


### getAngleWithTargetVectorColor

```
private java.awt.Color getAngleWithTargetVectorColor()
```

---


### getAngleEnergyColor

```
private java.awt.Color getAngleEnergyColor()
```

---


### getHeadOrTailColor

```
private java.awt.Color getHeadOrTailColor()
```

---


### getCell

```
public ProtrusiveCell getCell()
```

:   **Overrides:**: `getCell` in class `Pixel`

---


### setAsCenter

```
protected void setAsCenter(boolean pOn)
```

---


### getAngleWithTargetVector

```
public double getAngleWithTargetVector()
```

---


### getAngleWithTargetVector

```
public double getAngleWithTargetVector(ProtrusiveCell c)
```


---


|  |  |  |
| --- | --- | --- |
| **Overview** \| **Package** \| **Class** \| **Use** \| **Tree** \| **Deprecated** \| **Index** \| **Help** | |  |
| **PREV CLASS**   **NEXT CLASS** | **FRAMES**    **NO FRAMES**     **All Classes** |  |
| SUMMARY: NESTED \| FIELD \| CONSTR \| METHOD | DETAIL: FIELD \| CONSTR \| METHOD |  |


---
